# Letter to the Editor: Reduced intraepidermal nerve fiber density after a sustained increase in insular glutamate: a proof-of-concept study examining the pathogenesis of small fiber pathology in fibromyalgia

**DOI:** 10.1097/PR9.0000000000000733

**Published:** 2019-04-15

**Authors:** Xavier J. Caro, Earl F. Winter

**Affiliations:** Southern California Fibromyalgia Research & Treatment Center, Northridge Hospital Medical Center, Northridge, CA, USA.

Gentlemen: We wish to comment on the recent article by Harte et al.,^6^ and its implications for research in, and the understanding of, fibromyalgia (FM). In their study, the authors report a significantly diminished intraepidermal nerve fiber density (IENFD) in rats whose central nervous systems (CNSs) had been infused, over 6 weeks, with L-trans-pyrrolidine-2, 4-dicarboxylic acid (PDC), a compound known to increase CNS levels of glutamate. They note that FM is known to be associated with increased CNS levels of glutamate,^5,15^ and, thus, conclude that their findings show that increased CNS glutamate, in and of itself, is associated with reduced IENFD. They reason that the reduced and injured IENFD reported in FM^3,8,11^ is, therefore, likely to be an epiphenomenon because of increased CNS glutamate. They purport that their findings represent a “proof of [this] concept.” Despite the authors' arguments, we do herein respectfully question their conclusions.

The authors have overlooked the potential role of a second relevant variable in their research design; that is, the systemic effect of the cranial surgery itself on these rodents. It has recently been demonstrated, for example, that humans admitted to an intensive care unit for traumatic brain injury (TBI) sustain a rather rapid onset peripheral neuropathic injury, including reduction in IENFD, ie, small fiber neuropathy.^10^ This small fiber neuropathy may be seen within days of admission and likely worsens over the ensuing weeks of their intensive care unit stay.^13^ It has been reported that such a TBI leads to both local and systemic immune activation including a low-grade cytokinopathy.^7,9^ Such an elevation in cytokines has been shown to correlate with reduced IENFD in FM.^2^ We certainly see no reason that this biologic phenomenon would not translate into the physiology of the rat model used by the authors.

The authors' choice of a craniotomy-naive rat cohort for statistical comparison with their craniotomy-PDC–treated rats' IENFD appears, therefore, to be an unfortunate methodological choice. Even if the reader accepts that statistical analysis demonstrated a significant difference between the naive and PDC2-treated groups' IENFD (Fig. 2; *P* = 0.047), a problem remains. The trouble with this statistical result is that although the PDC2 group has undergone both surgery and bilateral PDC infusion, the naive group has undergone neither of these insults. Instead, comparison of the authors' craniotomy-PDC–infused (unilateral “PDC1” and bilateral “PDC2”) rat groups with their craniotomy-Ringer's solution–infused rat group seems a more appropriate IENFD control. This latter group was subjected to the same cranial trauma that their PDC-treated rat groups were, thus controlling for the effect of this surgical insult to the CNS. By their own statistical analysis, the authors report no significant differences in IENFD between these 3 study groups (Fig. 2).

Furthermore, interpretation of the authors' data is hindered by the small number of observations within each of their experimental cohorts. This is particularly apparent in their analysis of the PDC2 group (N = 4), and when they conclude that the IENFD in a single rat (IENFD = 15.15 fibers/mm), whose microinjectors were “outside the insula,” suggested to them that the, “PDC effect may exhibit anatomical specificity to the insula.” We contend that an analysis of such small samples, including that of a single data point, is difficult and questionable at best.

We also have concerns for the authors' observation that there were no substantial differences in the morphological features of the cytoarchitecture within the brain regions of the rats infused with PDC (Fig. 3). Our perusal of the “INS” (ie, insula)-labeled photomicrographs from the Ringer-treated and the PDC-treated groups (Fig. 3B) suggests a visible difference in the apparent morphology of this delicate tissue (evaluation with iMac Pro; resolution 5120 × 2880 pixels). Of course, we understand the potential difficulties in our judging published, web-based photomicrographs. We would merely suggest that the authors might have considered reporting the results of a controlled, blinded, systematically scored tabulation of the tissue slices used to grade and detect any such brain cytoarchitectural disruption, thus strengthening their “glutamate has done no harm” contention.

Any apparent injury pattern in brain tissue would not simply be of academic interest. PDC infusion, as described by the authors, would be expected to flood the insula with glutamate, a known neuroexcitotoxin that might easily have led to important functional and structural changes in this delicate tissue.^1,14^ This flooding would come atop a hyperglutaminergic tide already produced by the TBI itself.^14^

The authors’ observation of enhanced hind paw nociception for mechanical and thermal, but not cold, stimuli in their rats parallels that seen by others using the lateral fluid percussion model of TBI.^4.12^ In that experimental model, lateral trephination of the calvarium is followed by a measured fluid, micro-hammer insult delivered to the rat brain without violating its blood–brain barrier, an injury typically followed by contralateral, sooner than ipsilateral, mechanica hyperalgesia, and thermal or cold allodynia. The only obstacle to invoking such an injury pattern to the authors' experiment is the seeming absence of a direct CNS injury, although broaching the dura mater and disruption of the blood–brain barrier might be considered an additive insult. Thus, our concern that PDC infusion, and its evoked hyperglutaminergic state, acts to produce a significant lesion in the rodent brain.

We conclude, therefore, that the authors have demonstrated remarkable ingenuity in studying FM in an animal model. They have, however, apparently overlooked important and relevant variables in their research design and data analysis, particularly the potential role of head trauma and PDC itself. We suggest that future study of their animal model, with these variables kept in mind, may show that PDC infusion, and consequent glutamate flooding of the rat CNS, at physiologic or nontoxic concentrations, is not—in and of itself—directly associated with reduction in IENFD, but likely mediates this effect via other well-described associated phenomena, such as activation of a local and systemic immune reaction and subsequent inflammatory response in the rat's brain and peripheral nervous system.

## Disclosures

The authors have no conflict of interest to declare.
